# Serum Cell Death Biomarkers for Prediction of Liver Fibrosis and Poor Prognosis in Primary Biliary Cirrhosis

**DOI:** 10.1371/journal.pone.0131658

**Published:** 2015-06-25

**Authors:** Tomohiro Sekiguchi, Takeji Umemura, Naoyuki Fujimori, Soichiro Shibata, Yuki Ichikawa, Takefumi Kimura, Satoru Joshita, Michiharu Komatsu, Akihiro Matsumoto, Eiji Tanaka, Masao Ota

**Affiliations:** 1 Department of Medicine, Division of Hepatology and Gastroenterology, Shinshu University School of Medicine, Matsumoto, Japan; 2 Department of Legal Medicine, Shinshu University Hospital, Matsumoto, Japan; Emory University School of Medicine, UNITED STATES

## Abstract

The development of simple, noninvasive markers of liver fibrosis is urgently needed for primary biliary cirrhosis (PBC). This study examined the ability of several serum biomarkers of cell death to estimate fibrosis and prognosis in PBC. A cohort of 130 patients with biopsy-proven PBC and 90 healthy subjects were enrolled. We assessed the utility of the M30 ELISA, which detects caspase-cleaved cytokeratin-18 (CK-18) fragments and is representative of apoptotic cell death, as well as the M65 and newly developed M65 Epideath (M65ED) ELISAs, which detect total CK-18 as indicators of overall cell death, in predicting clinically relevant fibrosis stage. All 3 cell death biomarkers were significantly higher in patients with PBC than in healthy controls and were significantly correlated with fibrosis stage. The areas under the receiver operating characteristic curve for the M65 and M65ED assays for differentiation among significant fibrosis, severe fibrosis, and cirrhosis were 0.66 and 0.76, 0.66 and 0.73, and 0.74 and 0.82, respectively. In multivariate analysis, high M65ED (hazard ratio 6.13; 95% confidence interval 1.18–31.69; *P* = 0.031) and severe fibrosis (hazard ratio 7.45; 95% confidence interval 1.82–30.51; *P* = 0.005) were independently associated with liver-related death, transplantation, or decompensation. High serum M65ED was also significantly associated with poor outcome in PBC (log-rank test; *P* = 0.001). Noninvasive cell death biomarkers appear to be clinically useful in predicting fibrosis in PBC. Moreover, the M65ED assay may represent a new surrogate marker of adverse disease outcome.

## Introduction

Primary biliary cirrhosis (PBC) is a chronic autoimmune cholestatic liver disease characterized by portal inflammation and immune-mediated destruction of intrahepatic bile ducts that often leads to cirrhosis and liver failure.[1] As severe fibrosis and cirrhosis are major risk factors for disease progression, the assessment of fibrosis is essential for adequate management of patients with PBC. Liver biopsy remains the gold standard for disease staging, but is limited by sampling error and the risk of complications.[2, 3] Accordingly, several noninvasive biomarkers have been developed to predict the degree of fibrosis.

In advanced biliary disease, apoptosis contributes to duct loss and is induced by signals such as activation of death receptors, immune-mediated injury, oxidative stress, infection, and toxins.[4] Apoptosis also promotes fibrogenesis, with apoptotic debris triggering the activation of hepatic stellate cells. Several other forms of cell death have been described in biliary disease, including necrosis, necroptosis, and autophagic cell death.[5–7] Both apoptosis and necrosis have been proposed to be responsible for the development and progression of liver fibrosis.[4, 8]

Various caspases are activated during apoptosis in PBC. In particular, cytokeratin-18 (CK-18), which represents a major intermediate filament protein in hepatocytes, is cleaved by caspases at 2 conserved aspartate residues. Bantel *et al*.[9] have developed a novel ELISA based on the M30 antigen that selectively recognizes an exposed neoepitope after cleavage of the CK-18 caspase substrate. Another assay, M65, detects both caspase-cleaved and uncleaved CK-18 and is therefore used as a marker of overall cell death from either apoptosis or necrosis.[10] Serum levels of CK-18 fragments are increased in various acute and chronic liver diseases.[10–13] Moreover, several studies have shown that CK-18 fragments can accurately differentiate nonalcoholic steatohepatitis from nonalcoholic fatty liver disease,[14–17] although the results are controversial.[18–21] The M65 assay is based on capture (M6) and detection (M5) antibodies directed against 2 different epitopes of CK-18 and therefore recognizes total CK-18. Although the newly developed M65 Epideath (ED) ELISA also represents total cell death, it uses the M6 antibody for detection and M5 as the capture antibody, resulting in improved binding specificity and lower signals in healthy controls.[22] To date, reports have been scarce regarding CK-18 fragments in PBC. Denk *et al*.[23] observed that CK-18 fragment levels were elevated in patients with PBC. However, no histological findings were assessed and only 52 patients were investigated. The objective of this study was to evaluate the ability of the CK-18-related M30, M65, and M65ED assays in predicting clinically relevant fibrosis stage and prognosis in PBC patients.

## Materials and Methods

### Subjects

The general demographic, clinical, and biochemical features of the 130 Japanese PBC patients who were enrolled in this study between January 1989 and December 2014 are summarized in Table 1. All patients were treated with ursodeoxycholic acid and followed for at least 6 months. The data of 90 sex- and age-matched healthy Japanese subjects were adopted as controls.PBC diagnosis was based on the criteria of the American Association for the Study of Liver Diseases.[24] All laboratory data were obtained on the same day as the liver biopsy. Alanine aminotransferase (ALT), aspartate aminotransferase (AST), alkaline phosphatase (ALP), γ-glutamyltransferase (GGT), and other relevant biochemical tests were performed using standard methods.[25] Serum anti-mitochondrial antibody (AMA) was determined using indirect immunofluorescence, whereby a titer of ≥1:40 was considered to be positive.[26] All subjects were negative for the hepatitis B surface antigen, antibody to hepatitis B core antigen, antibody to hepatitis C virus, and antibody to the human immunodeficiency virus. The study was conducted according to the guidelines of the Declaration of Helsinki and was approved by the ethics committee of Shinshu University School of Medicine. Written informed consent was obtained from all subjects. Serum samples were obtained at the time of liver biopsy prior to ursodeoxycholic acid treatment and stored unthawed in several tubes at -30°C until testing.

**Table 1:** Demographic and Clinical Characteristics of Patients with PBC at Different Stages of Fibrosis.

Characteristic	All subjects (n = 130)	F0-1 (n = 81)	F2-3 (n = 44)	F4 (n = 5)	*P* Value
Age (years)	57 (49–63)	57 (51–62)	58 (50–65)	48 (48–59)	0.727
Female, n (%)	111 (85)	68 (84)	39 (89)	4 (80)	0.733
AMA-positive, n (%)	110 (85)	63 (78)	42 (96)	5 (100)	0.013
Bilirubin (mg/dL)	0.8 (0.6–1.0)	0.7 (0.5–0.9)	0.8 (0.6–1.0)	1.6 (1.4–8.2)	0.001
ALT (IU/L)	42 (28–69)	36 (27–57)	55 (33–88)	71 (58–105)	0.008
AST (IU/L)	40 (30–61)	36 (29–53)	53 (34–92)	91 (81–154)	<0.001
ALP (IU/L)	439 (325–605)	402 (297–572)	488 (378–738)	1921 (184–2250)	0.048
GGT (IU/L)	136 (86–261)	114 (72–206)	192 (116–286)	230 (209–322)	0.008
Albumin (g/dL)	4.2 (4.0–4.5)	4.3 (4.2–4.5)	4.1 (3.9–4.4)	3.1 (2.9–3.6)	<0.001
M30 (U/L)	381 (229–586)	331 (230–461)	421 (277–825)	372 (241–835)	0.052
M65 (U/L)	658 (387–1260)	593 (350–1043)	1069 (401–1676)	1075 (860–1778)	0.006
M65ED (U/L)	672 (485–1278)	553 (466–753)	1158 (683–1700)	1565 (1257–1960)	<0.001

Data are presented as the median (25^th^-75^th^ percentile) for continuous variables and total number (%) for categorical variables.

*P* values correspond to a comparison of the 3 subject groups.

### Liver histology

Liver biopsy was performed by percutaneous sampling of the right lobe with a 14-gauge needle in all but 4 patients before administration of ursodeoxycholic acid. All biopsy samples were 1.5 cm or more in length. Formalin-fixed and paraffin-embedded specimens were prepared and used for histopathological studies. Sections measuring 4 μm were cut from each paraffin block and stained with hematoxylin and eosin, periodic acid-Schiff after diastase digestion, and Azan-Mallory. Liver fibrosis and necroinflammatory activity were evaluated according to the METAVIR scoring system:[27] significant fibrosis was defined as ≥F2, severe fibrosis as ≥F3, and cirrhosis as F4.

### Detection of total and caspase-cleaved CK-18

Caspase-generated CK-18 fragment levels were measured in all serum samples using the M30-Apoptosense ELISA (Pevia, Bromma, Sweden), which was designed to specifically detect apoptotic CK-18-positive cells in human serum and quantitatively measure the apoptosis-associated neoepitopes in the C-terminal domain of CK-18 (amino acids 387–396).[9] We also adopted the M65 and improved M65ED ELISAs (Pevia), which quantify both caspase-cleaved and uncleaved CK-18.[10] All assays were performed under blinded conditions.

### Statistical analysis

Categorical variables were compared using Pearson’s chi-square or Fisher’s exact test and continuous variables were analyzed with the Mann-Whitney *U* or Kruskal-Wallis test, as appropriate. Spearman’s rank order correlations were employed to evaluate associations between serum cell death markers and clinical features. The diagnostic performance of each marker was determined in terms of sensitivity, specificity, positive predictive value (PPV), negative predictive value (NPV), and area under the receiver operating characteristic (ROC) curve (AUC). The selection of optimal cutoff values was based on the Youden index. Multiple logistic regression models were adopted to identify factors predictive of significant fibrosis. Factors attaining a *P* < 0.1 in univariate analysis were used for ensuing multivariate analysis. Clinical outcome as of December 2014 was recorded as liver-related death, liver transplantation, or liver decompensation (i.e., ascites, hepatocellular carcinoma, or hepatic encephalopathy). Kaplan-Meier curves were used to analyze the survival rates of patients using the log-rank test. Cox regression multivariate analysis (forward stepwise likelihood-quotient) was performed to predict survival rates. All analyses were performed using IBM SPSS Statistics version 21.0 software (IBM, Chicago, IL). A *P* < 0.05 was considered to be statistically significant.

## Results

### Patient characteristics

The basic demographic, clinical, and biochemical features of the 130 PBC patients are shown in Table 1. Median age was 57 years, and 111 (85%) subjects were female. Serum bilirubin, ALT, AST, and GGT levels were significantly higher, and serum albumin was significantly lower, in patients with cirrhosis. Fibrosis stage was F0 in 10 cases (8%), F1 in 71 cases (55%), F2 in 27 cases (21%), F3 in 17 cases (13%), and F4 in 5 cases (4%).

### Cell death markers and fibrosis stage in PBC

**Fig 1:**
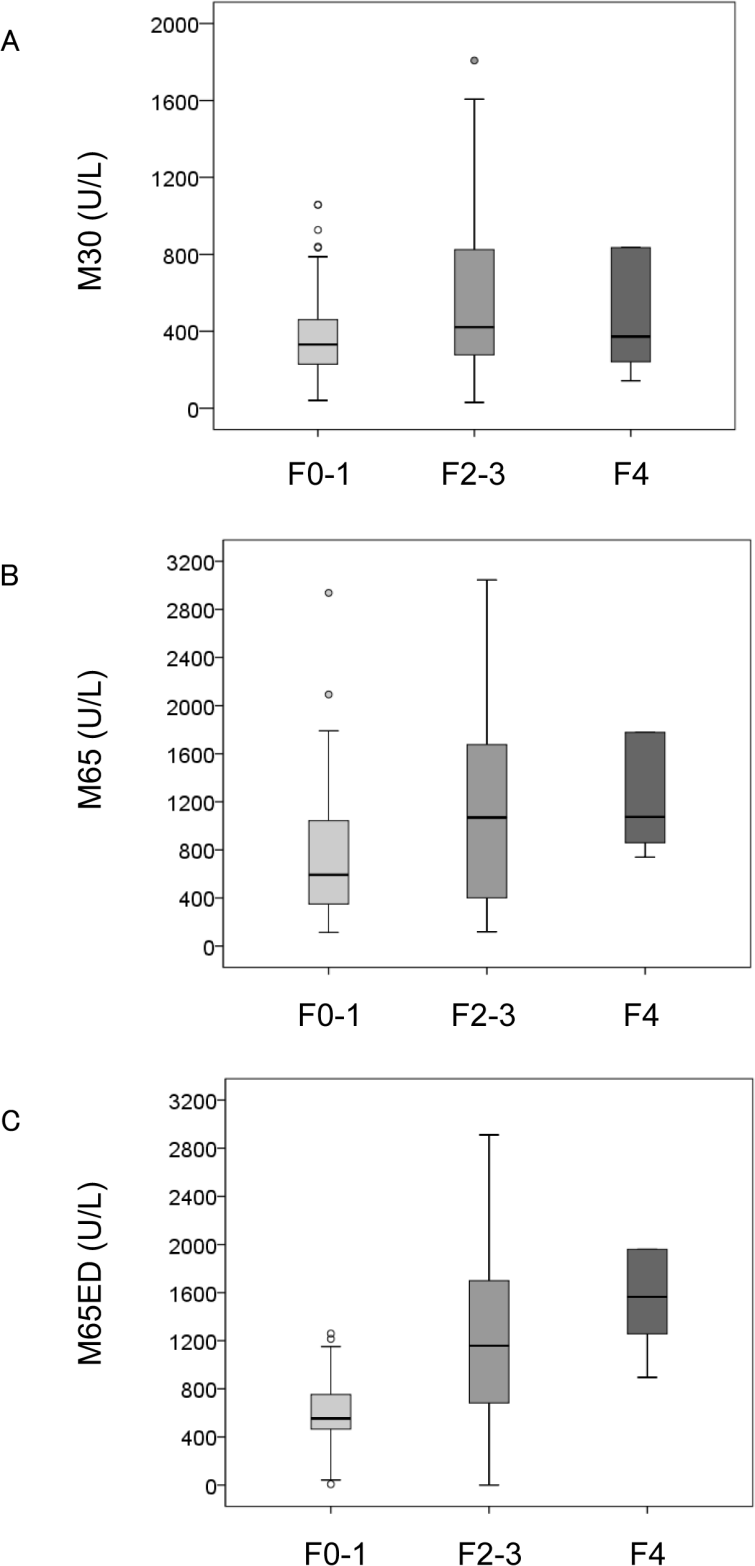
Correlation of the cell death biomarkers (A) M30, (B) M65, and (C) M65ED with liver fibrosis scores in primary biliary cirrhosis. Boxes represent the interquartile range (IQR) of the data. The lines across the boxes indicate the median values. Hash marks depict the nearest value within 1.5 times the IQR. Open circles indicate outliers. Detection by M30 (A) and M65 (B) was unable to significantly discriminate between F0-F3 and F4 fibrosis stages but could differentiate between F0-1 and ≥F2 or F0-2 and ≥F3 stages of fibrosis. Estimation using M65ED could significantly discriminate between all fibrosis stages.

The median serum levels of the M30, M65, and M65ED ELISAs (M30: 381 U/L vs. 62 U/L; *P* < 0.001, M65: 658 U/L vs. 60 U/L; *P* < 0.001, and M65ED: 672 U/L vs. 151 U/L; *P* < 0.001) were significantly higher in patients with PBC than in healthy subjects. The values of all 3 biomarkers (M30: 421 U/L vs. 331U/L; *P* = 0.016, M65: 1075 U/L vs. 593 U/L; *P* = 0.002, and M65ED: 1218 U/L vs. 553 U/L; *P* < 0.001) were significantly higher in patients with significant fibrosis (≥F2) than in those without (Fig 1). Multivariate analysis showed that albumin (odds ratio [OR]: 0.214, 95% confidence interval [CI]: 0.061–0.749), and M65ED (OR: 1.001, 95% CI: 1.000–1.001) were independently associated with the presence of significant fibrosis (Table 2). Similar findings were observed for patients with severe fibrosis (≥F3) (M30: 429 U/L vs. 370 U/L; *P* = 0.113, M65: 1144 U/L vs. 627 U/L; *P* = 0.019, and M65ED: 1506 U/L vs. 625 U/L; *P* = 0.001). Lastly, M65 (1075 U/L vs. 645 U/L; *P* = 0.070) and M65ED (1565 U/L vs. 651 U/L; *P* = 0.016) assay values were significantly higher in patients with cirrhosis (= F4) than in those without, but not those of M30 (384 U/L vs. 372 U/L; *P* = 0.218).

**Table 2:** Univariate and Multivariate Analyses of Parameters Associated with Significant Fibrosis (F2-4) in Liver Biopsy.

Parameter	F0-1 (n = 81)	F2-4 (n = 49)	Univariate *P* value	Multivariate OR (95% CI)	Multivariate *P* value
Age (years)	57 (51–62)	58 (48–65)	0.768		
Female, n (%)	68 (84)	43 (88)	0.552		
Bilirubin (mg/dL)	0.7 (0.5–0.9)	0.8 (0.6–1.1)	0.025		
ALT (IU/L)	36 (27–57)	57 (34–89)	0.002		
AST (IU/L)	36 (29–53)	53 (35–94)	<0.001		
ALP (IU/L)	402 (297–572)	495 (357–756)	0.014	1.002 (1.000–1.003)	0.057
GGT (IU/L)	114 (72–206)	209 (116–288)	0.002		
Albumin (g/dL)	4.3 (4.2–4.5)	4.1 (3.9–4.3)	0.001	0.214 (0.061–0.749)	0.016
M30 (U/L)	331 (229–461)	421 (255–825)	0.016		
M65 (U/L)	593 (350–1043)	1075 (478–1683)	0.002		
M65ED (U/L)	553 (466–753)	1218 (700–1785)	<0.001	1.001 (1.000–1.001)	0.014

Quantitative variables are expressed as median (interquartile range).

### Correlation between clinical characteristics and cell death markers

We examined demographic and clinical characteristics for correlations with the M30, M65, and M65ED assays in patients with PBC (Table 3). The M30 assay was significantly correlated with bilirubin and fibrosis stage. The M65 assay correlated significantly with bilirubin, ALT, AST, ALP, and fibrosis stage, while M65ED was significantly associated with bilirubin, ALT, AST, ALP, and fibrosis stage. All 3 assays were significantly inversely correlated with albumin.

**Table 3:** Correlation of M30, M65, and M65ED Levels with Demographic and Clinical Characteristics.

Characteristic	M30		M65		M65ED	
	rho	*P* value	rho	*P* value	rho	*P* value
Age (years)	0.05	0.553	-0.04	0.689	-0.07	0.452
Bilirubin (mg/dL)	0.22	0.011	0.43	<0.001	0.48	<0.001
ALT (IU/L)	0.10	0.257	0.29	0.001	0.24	0.007
AST (IU/L)	0.17	0.061	0.35	<0.001	0.31	<0.001
ALP (IU/L)	0.17	0.050	0.26	0.003	0.35	<0.001
GGT (IU/L)	0.05	0.56	0.08	0.341	0.10	0.258
Albumin (g/dL)	-0.24	0.007	-0.24	0.006	-0.26	0.003
Fibrosis	0.28	0.001	0.37	<0.001	0.36	<0.001

### Diagnostic value of cell death biomarkers for fibrosis stage

**Fig 2:**
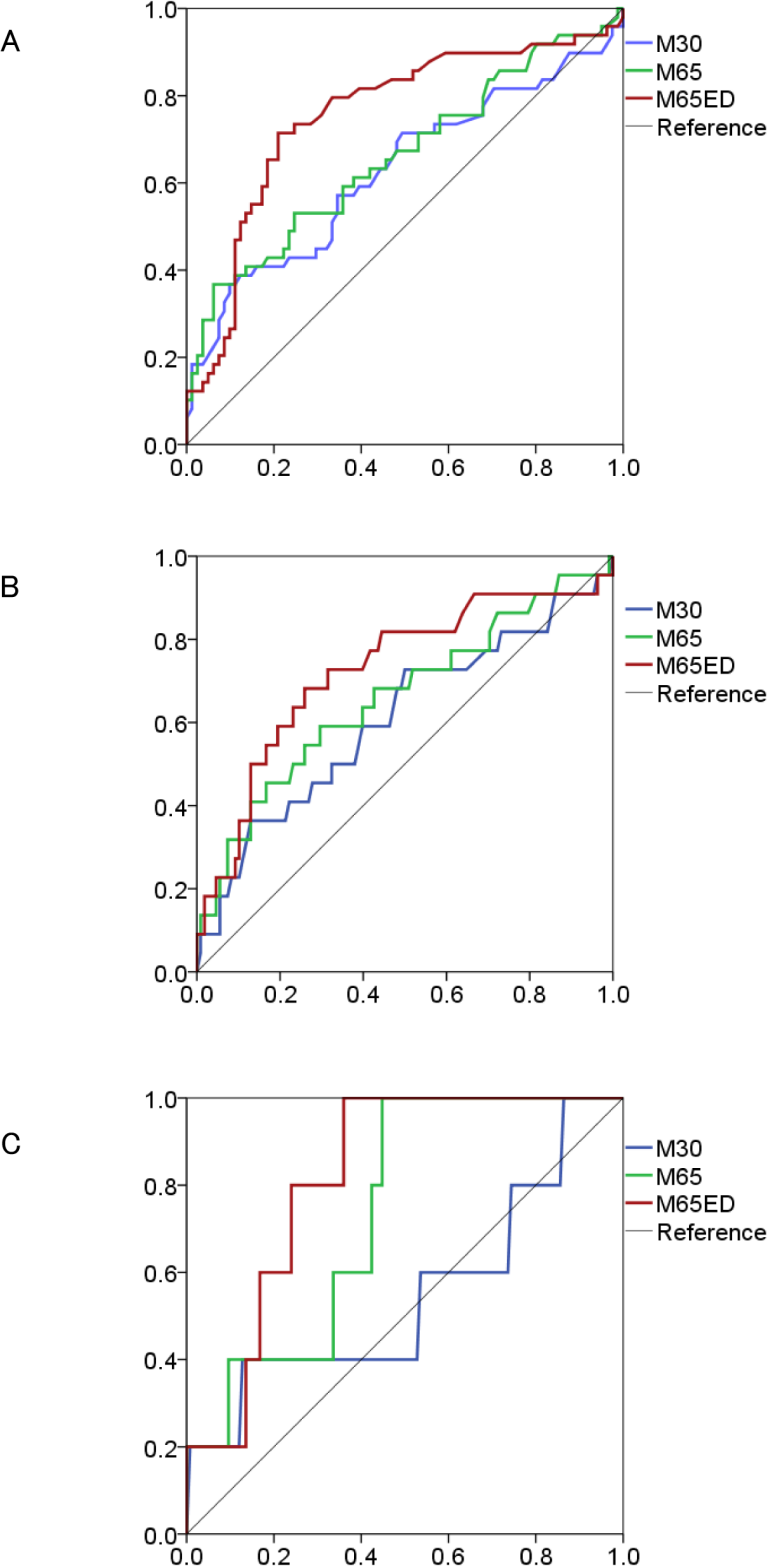
Receiver operating characteristic analysis of the 3 cell death biomarkers (M30, M65, and M65ED) for the prediction of (A) significant fibrosis (≥F2), (B) severe fibrosis (≥F3), and (C) cirrhosis (F4).

ROC analysis was performed to determine the optimal cutoff values for the cell death biomarker assays to distinguish among significant fibrosis (≥F2), severe fibrosis (≥F3), and cirrhosis (= F4) (Fig 2). The sensitivity, specificity, PPV, NPV, accuracy, and calculated AUC for each biomarker are listed in Table 4. Total CK-18 level detected by the M65ED ELISA of above or below 830 U/L correctly predicted significant fibrosis with a sensitivity of 71%, specificity of 79%, and AUC of 0.76 (95% CI: 0.67–0.85). The AUC values for the M30 and M65 assays were comparatively lower at 0.63 (95% CI: 0.52–0.73) and 0.66 (95% CI: 0.56–0.76), respectively. The AUCs of the M30, M65, and M65ED assays for predicting severe fibrosis were 0.61 (95% CI: 0.47–0.75), 0.66 (95% CI: 0.52–0.80), and 0.73 (95% CI: 0.60–0.85), respectively. The sensitivity/specificity of the M65ED assay was good at 68%/74%. Lastly, the M65 and M65ED assays provided similar discriminating power and good compromise of sensitivity/specificity to predict cirrhosis (M65: 100%/55%, AUC 0.74 [95% CI: 0.57–0.91] and M65ED: 100%/64%, AUC 0.82 [95% CI: 0.70–0.94]).

**Table 4:** Predictive Ability of the M30, M65, and M65ED Assays by Fibrosis Stage.

	M30	M65	M65ED
**Fibrosis stage ≥2**			
AUC (95% CI)	0.63 (0.52–0.73)	0.66 (0.56–0.76)	0.76 (0.67–0.85)
Sensitivity (%)	37	37	71
Specificity (%)	90	94	79
PPV (%)	69	78	67
NPV (%)	70	71	82
Accuracy (%)	70	72	76
**Fibrosis stage ≥3**			
AUC (95% CI)	0.61 (0.47–0.75)	0.66 (0.52–0.80)	0.73 (0.60–0.85)
Sensitivity (%)	36	59	68
Specificity (%)	87	70	74
PPV (%)	36	29	35
NPV (%)	87	89	92
Accuracy (%)	79	69	73
**Fibrosis stage = 4**			
AUC (95% CI)	0.55 (0.25–0.85)	0.74 (0.57–0.91)	0.82 (0.70–0.94)
Sensitivity (%)	40	100	100
Specificity (%)	87	55	64
PPV (%)	11	8	10
NPV (%)	97	100	100
Accuracy (%)	85	57	65

Cutoff values were determined using receiver operating characteristic (ROC) curves.

### Association between cell death biomarkers and adverse effects

The patients were followed for a median of 8.1 years (range: 0.5–25.3 years), during which time 11 (8%) adverse events (5 cases of liver-related death [2 from hepatocellular carcinoma and 3 from ruptured esophageal varices] and 3 cases each of liver transplantation and liver decompensation) occurred. In multivariate Cox regression analysis that included all factors identified to be associated with survival in the univariate analysis (GGT; *P* = 0.016, fibrosis; *P* = 0.001, M65ED; *P* = 0.004, M65; *P* = 0.031), M65ED ≥ 672 U/L (hazard ratio 6.13; 95% CI: 1.18–31.71; *P* = 0.031) and severe fibrosis (F3 or F4) (hazard ratio 7.66; 95% CI: 1.87–31.34; *P* = 0.005) were identified as independent variables associated with poor prognosis.

**Fig 3:**
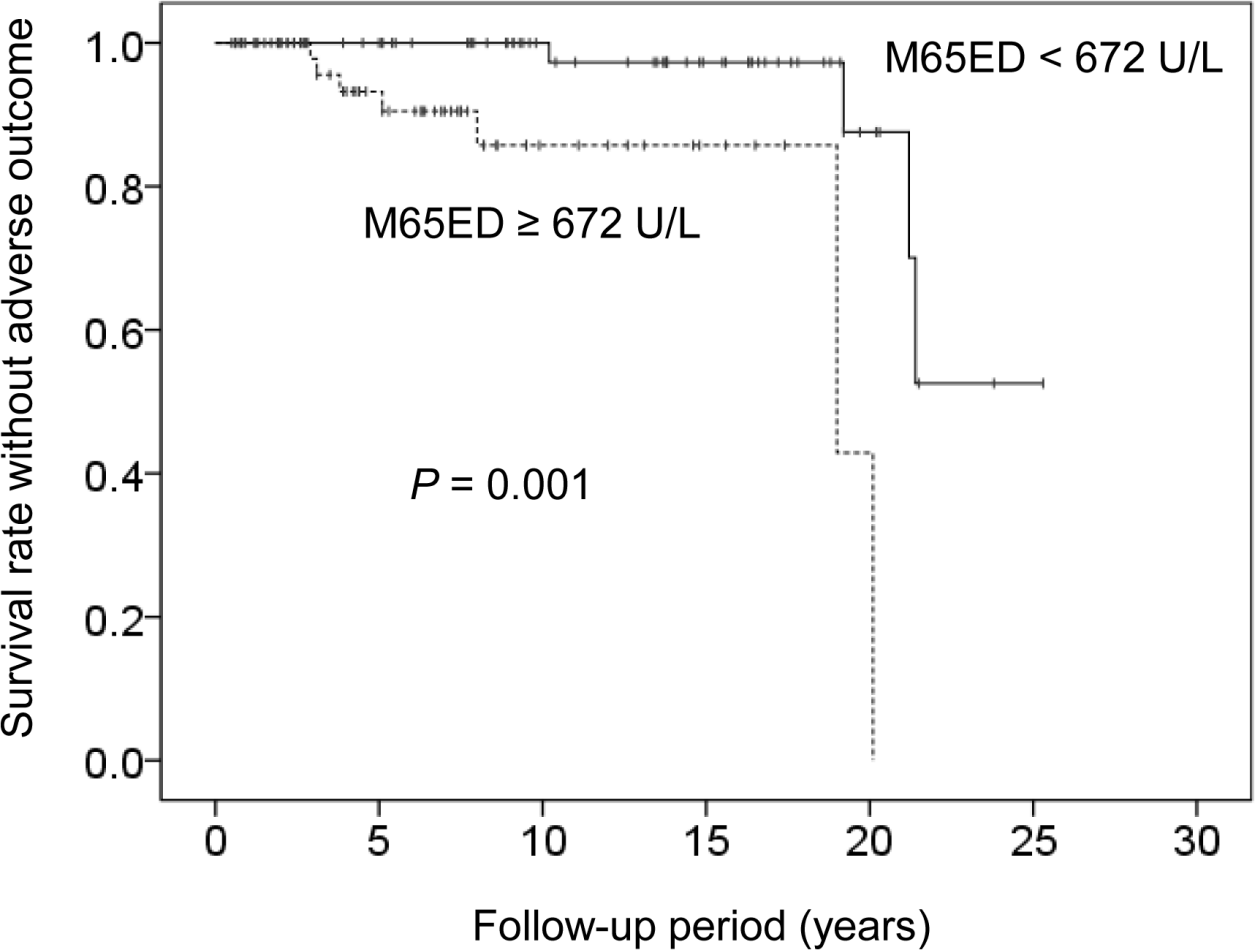
Cumulative survival rate analyzed using the Kaplan-Meier method according to baseline M65ED levels. Survival was significantly lower in patients when M65ED level was ≥ 672 U/L (*P* = 0.001).

Kaplan-Meyer analysis of overall survival was performed using the median serum M65ED level (672 U/L) at diagnosis as the cut-off (Fig 3). The cumulative survival rate without an adverse outcome between the groups was significantly different (log-rank test; *P* = 0.001).

## Discussion

PBC is a serious condition with a high risk of liver failure and liver-related mortality that typically progresses to cirrhosis. The prognosis of patients with PBC appears to be dictated by the presence and extent of fibrosis in liver biopsy findings. Until now, liver biopsy has been the gold standard for assessing fibrosis. However, the utility of liver biopsy is limited by sampling error, inter- and intra-observer discrepancies, risk of complications, and the limited frequency of examinations.[2, 3] Moreover, there is considerable variation in the degree of fibrosis throughout the liver of PBC patients; in one study, fibrosis not detected in small needle biopsy specimens was regularly witnessed in hepatectomy samples obtained at transplantation, and only 36% of hepatectomy samples were concordant with the predicted degree of fibrosis.[28] Furthermore, a discrepancy of 1 or 2 fibrosis stages was found in 64% of cases when later examined by a simulated liver needle biopsy method. These findings indicate an urgent need for simple, sensitive/specific, noninvasive tests to estimate fibrosis stage in patients with PBC.

Hepatocellular death is classically viewed to occur by either programmed apoptosis or accidental, uncontrolled cell death (i.e., necrosis).[6, 7] Hepatocyte death is the key trigger of liver disease progression, which is characterized by inflammation, and fibrosis. In the present study, we examined 3 cell death biomarkers in patients with PBC and analyzed their association with liver histology. Caspase-cleaved CK-18 fragments (M30 assay) and total CK-18 (M65 assays) have already been correlated with fibrosis stage in several chronic liver diseases.[9–11, 14, 15] Similarly, we found a significant correlation between M30, M65, and M65ED levels and histological fibrosis PBC. All assays could differentiate between significant and mild fibrosis. Moreover, the M65ED assay provided better diagnostic accuracy for the detection of significant fibrosis (≥F2) compared with the M30 and M65 assays and was independently associated with the presence of significant fibrosis in multivariate analysis. Recently, serum *Wisteria floribunda* agglutinin-positive Mac-2-binding protein was reported as a novel, noninvasive method of estimating liver fibrosis in PBC that was well correlated with fibrosis stage.[29] Clinical validation of cell death and other biomarkers is needed in future large-scale trials. The M65 and M65ED assays showed similarly high AUCs in predicting liver cirrhosis. Since it is difficult to perform liver biopsy in patients with cirrhosis, the number of cirrhosis cases was small in the present study. However, this may underscore the need to establish noninvasive fibrosis markers in PBC.

Increased cholangiocyte apoptosis in the presence of activated cytotoxic T cells is evident in PBC,[30–32] in which autoantibodies are specific for a conformation-dependent epitope of the E2 subunit of the pyruvate dehydrogenase complex (PDC-E2), a ubiquitous mitochondrial membrane component. Odin et al.[33] observed that PDC-E2 was neither cleaved by caspases nor concentrated into surface blebs in apoptotic cells. The expression of BCL-2 and depletion of glutathione before the induction of apoptosis prevented this loss of autoantibody recognition, suggesting that glutathiolation of PDC-E2 was responsible for an absence in immunofluorescence signaling. Apoptotic cholangiocytes may therefore be a potential source of immunogenic PDC-E2 in PBC. Since BCL-2 expression was also found in hepatocytes in PBC,[31] CK-18 fragments were believed to reflect hepatocyte apoptosis.

There exists another study in which all 3 cell death biomarkers were examined in non-alcoholic steatohepatitis.[20] The specificity of M30, M65, and M65ED was 61%, 67%, and 68%, respectively, to discriminate significant fibrosis and 71%, 78%, and 77%, respectively, to estimate progressed fibrosis/cirrhosis. Although the specificities uncovered for PBC are higher in the present report, direct comparisons are limited by differences in disease etiology and patient ethnicity.

It was recently shown that M65 could predict survival in patients with acute liver failure.[34] Furthermore, lower serum M65 levels have been associated with longer overall survival in hepatocellular carcinoma.[35] Here, higher M65ED, but not M30, levels correlated with a poor outcome, implying that the assessment of necrotic hepatocyte death could be a suitable prognostic marker as well.

This study has several limitations. First, bias might have been included during case selection because the clinical indications for liver biopsy were unclear. Second, since patients with advanced fibrosis tended not to undergo liver biopsy, the proportions of subjects with severe fibrosis or cirrhosis were small in this study, which likely widened the confidence intervals of the 3 cell death biomarkers.

In conclusion, our findings demonstrate that the measurement of total CK-18 by the M65 and M65ED ELISAs, and to a lesser extent caspase-cleaved CK-18 by M30, may enable determination of fibrosis stage in patients with PBC. In particular, elevated levels of M65ED might also be associated with poor prognosis in PBC. Further studies are needed to confirm the usefulness of these biomarkers in clinical practice as noninvasive indicators of PBC.
